# T-Bet Controls Cellularity of Intestinal Group 3 Innate Lymphoid Cells

**DOI:** 10.3389/fimmu.2020.623324

**Published:** 2021-02-02

**Authors:** Jan-Hendrik Schroeder, Katrin Meissl, Dominika Hromadová, Jonathan W. Lo, Joana F. Neves, Jane K. Howard, Helena Helmby, Nick Powell, Birgit Strobl, Graham M. Lord

**Affiliations:** ^1^ School of Immunology and Microbial Sciences, King’s College London, London, United Kingdom; ^2^ Institute of Animal Breeding and Genetics, University of Veterinary Medicine Vienna, Vienna, Austria; ^3^ Division of Digestive Diseases, Faculty of Medicine, Imperial College London, London, United Kingdom; ^4^ Centre for Host-Microbiome Interactions, King’s College London, London, United Kingdom; ^5^ Department of Diabetes, School of Life Course Sciences, Faculty of Life Sciences and Medicine, King’s College, London, United Kingdom; ^6^ Department of Infection Biology, London School of Hygiene and Tropical Medicine, London, United Kingdom; ^7^ Faculty of Biology, Medicine and Health, University of Manchester, Manchester, United Kingdom

**Keywords:** T-bet, innate lymphoid cells, ILCs, intestinal inflammation, mucosal homeostasis

## Abstract

Innate lymphoid cells (ILC) play a significant immunological role at mucosal surfaces such as the intestine. T-bet-expressing group 1 innate lymphoid cells (ILC1) are believed to play a substantial role in inflammatory bowel disease (IBD). However, a role of T-bet-negative ILC3 in driving colitis has also been suggested in mouse models questioning T-bet as a critical factor for IBD. We report here that T-bet deficient mice had a greater cellularity of NKp46-negative ILC3 correlating with enhanced expression of RORγt and IL-7R, but independent of signaling through STAT1 or STAT4. We observed enhanced neutrophilia in the colonic lamina propria (cLP) of these animals, however, we did not detect a greater risk of T-bet-deficient mice to develop spontaneous colitis. Furthermore, by utilizing an *in vivo* fate-mapping approach, we identified a population of T-bet-positive precursors in NKp46-negative ILC3s. These data suggest that T-bet controls ILC3 cellularity, but does do not drive a pathogenic role of ILC3 in mice with a conventional specific pathogen-free microbiota.

## Introduction

CD127^+^ innate lymphoid cells (ILC) have been categorized into subsets based on expression of characteristic transcription factors (1). ILC1 express T-bet (encoded by *Tbx21*). ILC2 express GATA3 while ILC3 have a characteristic expression of RORγt. The ILC3 group has three members depending on the expression of NKp46 and CCR6. NKp46^+^ ILC3 express T-bet, and CCR6^-^ NKp46^-^ ILC3 have been suggested to be the precursors of these cells (2). In contrast CCR6^+^ ILC3 do not express T-bet and are generated following a developmental pathway that is very distinct to the other ILC (3).

There is evidence associating IBD and colitis with T-bet-expressing cells alike ILC1. We have recently reported that enhanced functionality of T-bet variants among individuals is associated with Crohn’s disease and ulcerative colitis (4). Furthermore, IFNγ appears to be the most critical factor driving colitis while IL-17A and IL-13 play a less important role (5–8). Interestingly, these observations are corroborated by the observation that ILC1 are particularly abundant among the total amount of ILC in inflamed intestinal lamina propria of Crohn’s disease patients (9–11). Furthermore, the generation of ILC1 from an NKp46^+^ ILC3 source has been linked to colitis development (12). We have recently observed that NKp46-dependent deletion of *Tbx21* in mice leads to milder dextran sodium sulphate (DSS)-induced colitis, and this may be caused by the depletion of T-bet expressing ILC1 and ILC3 (13).

However, in contrast to these data supporting a critical pathogenic role of T-bet^+^ ILC in colitis, we have also reported previously that germline depletion of *Tbx21* in Rag-deficient BALB/c-background mice can trigger spontaneous colitis in the presence of and depending on *Helicobacter thyphlonius* as part of the microbiota. In these mice an elevated frequency of colitogenic IL-17A^+^ CD90^+^ CD127^+^ ILC within the lamina propria appeared to be the driving force of the pathological phenotype. A pathogenic role of ILC3 in colitis has also been suggested by others in mouse models with *H. hepaticus* and anti-CD40 antibody driven colitis and even IBD patients (7, 14, 15). Furthermore, CCR6^+^ ILC3 have been linked to enhanced airway hyperreactivity in an obesity model (16). In contrast to these pathogenic roles of ILC3, several studies highlight their protective functionality in the intestine [(17–26) reviewed by (27)]. As such ILC3 were reported to be a critical source of protective IL-22 early during infection with *Citrobacter rodentium*, and depletion of CD4^+^ CCR6^+^ ILC3 using an anti-CD4 antibody led to diminished IL-22 production and enhanced bacterial tissue infiltration (28, 29).

Hence, in immunocompetent patients, T-bet has the potential to be a potent drug target to control Crohn’s disease and ulcerative colitis, but it needs to be established whether this approach leads to an enhanced pathogenic role of ILC3. Encouragingly, *Tbx21* deficient mice on a BALB/c background do not develop spontaneous colitis (30). We have also reported previously that T-bet germline depletion in C57BL/6-background mice leads to an enhanced cellularity and functionality of ILC2 which may have a beneficial role to protect from colitis with a T-bet targeting treatment (13).

In this study, we report that *Tbx21* deficiency leads to greater cellularity of intestinal NKp46-negative ILC3 in Rag-sufficient and deficient mice. Interestingly, *Tbx21* deficiency in these cells caused enhanced expression of IL-7R and RORγt which may play a mechanistic role in driving a greater ILC3 cellularity in immunocompetent mice. In contrast, signaling events *via* STAT1 and STAT4 were not critically important to drive the enhanced cellularity of T-bet-negative ILC3. Despite the greater ILC3 cellularity, *Tbx21*-deficient mice did not develop spontaneous colitis. We believe this work highlights the critical role of T-bet to restrain ILC3 and neutrophil cellularity in the colonic lamina propria and provides critical insight into the feasibility of drug-based targeting of T-bet as therapeutic strategy for colitis.

## Methods

### Animals

C57BL/6, *Tbx21* ^-/-^ (C57BL/6 background), *Rag2* ^-/-^ (BALB/c background), and *Il27RA* ^-/-^ (C57BL/6 background) mice were sourced commercially (all Charles River). *Ifng* ^-/-^ (C57BL/6 background) mice were a gift from Dr Anne O’Garra (The Francis Crick Institute, London). A colony of colitis-free TRnUC mice was generated from a descendant of the TRUC colony described previously (31). *Stat1* ^-/-^ (B6.129P2-Stat1tm1Dlv) (32) and *Stat4* ^-/-^ (C57BL/6J-Stat4em3Adiuj/J, purchased from The Jackson Laboratory) mice were housed under specific pathogen-free conditions according to Federation of European Laboratory Animal Science Associations (FELASA) guidelines. C57BL/6N and C57BL/6J mice were purchased from Janvier Labs and used as control mice for *Stat1* ^-/-^ and *Stat4* ^-/-^ mice respectively. *Rosa26* ^YFP/+^ (Jackson labs) mice were sourced commercially and bred with T-bet^cre/+^ mice to generate the T-bet^Cre/+^x*Rosa26* ^YFP/+^ (T-bet^FM^) mice.

#### Generation of T-bet^cre/+^ Mouse

To allow the expression of the Cre-recombinase under the expression of the T-bet endogenous promoter, a T-bet knock-in mouse was generated (GenOway, France). For this purpose, an IRES-Cre cassette was introduced downstream of the Stop codon of the T-bet gene, in the 3’UTR (**Figure 2A**). The genomic region of interest containing the murine *Tbx21* locus was isolated by PCR from 129Sv genetic background. PCR fragments were subcloned into the pCR4-TOPO vector (Invitrogen). The genomic clones (containing intron 1 to exon 6) were used to construct the targeting vector. Briefly, a 5.6-kb fragment comprising *Tbx21* exon 2 and 6 and a 1.6-kb fragment located downstream of the *Tbx21* exon 6 STOP codon were used to flank an IRES-Cre cassette (FRT site-PGK promoter-Neo cDNA-FRT site).

##### Screening of T-Bet–Targeted Embryonic Stem Cell Clones

The FseI-linearized targeting vector was transfected into C57BL/6 ES cells. Positive selection was started 48 h after electroporation, by addition of 200 μg/ml G418 (150 μg/ml active component; Life Technologies). Then, 275 resistant clones were isolated, amplified, and screened by PCR and further confirmed by Southern blot.

##### Generation of Chimeric Mice and Breeding Scheme

One floxed mutated *Tbx21* ES cell clone was microinjected into albino C57BL/6 strain (C57BL/6J-Tyrc-2J/J) blastocysts, and gave rise to male chimeras with a significant ES cell contribution (as determined by the percentage of light and dark patches on their coat). After mating with C57BL/6 CMV-Flp–expressing female mice to remove the FRT-flanked Neo cassette, offspring were genotyped by PCR and Southern blot to ensure removal of the Neo cassette. PCR and Southern blot screening conditions are available on request. The mosaic excised F1 mouse was mated with C57BL/6 WT mice to obtain a pure line of Cre-expressing T-bet knock-in mice: T-bet^cre/+^.

### Isolation of Intestinal Leukocytes

cLP and Peyer’s patch-free SI LP leukocytes were isolated using a published method (33). Briefly, the epithelium was removed by incubation in HBSS lacking Mg^2+^ or Ca^2+^ (Invitrogen) supplemented with EDTA and HEPES. The tissue was further digested in HBSS lacking Mg^2+^ or Ca^2+^ supplemented with 2% foetal calf serum (FCS Gold, PAA Laboratories), 0.5 mg/ml collagenase D, 10 μg/ml DNase I, and 1.5 mg/ml dispase II (all Roche). The LP lymphocyte-enriched population was harvested from a 40–80% Percoll (GE Healthcare) gradient interface. For neutrophil analyses leukocytes were not purified by Percoll gradient centrifugation.

### Flow Cytometry

Flow cytometry was performed using a standard protocol. For ILC analyses a lineage cocktail of antibodies specific for CD3, CD45R, CD19, CD11b, TER-119, Gr-1, CD5, and FcϵRI was used. For a complete list of the antibodies used see **Table 1**. LIVE/DEAD^TM^ stain (ThermoFisher Scientific Inc.) was used to determine cell viability. A FoxP3 staining kit (ebioscience) was used for intracellular staining of transcription factors and cytokines. In case of cytokine analysis, cells were pre-stimulated with 100 ng/ml PMA and 2 µM ionomycin in the presence of 6 µM monensin for 3–4 h prior to flow cytometry analysis. Samples were acquired using an LSRFortessa™ cell analyser (Becton Dickinson, USA) or a Cytoflex LX™ for the data on *Stat1* ^-/-^ and *Stat4* ^-/-^ mice. All the data were analyzed using FlowJo software (Tree Star, USA). Cell counts were determined using a fixed amount of inclusion beads (Spherotec, Inc.) as a reference in the flow cytometry tubes.

**Table 1:** Antibody clones and distributors.

Antibody	Clone	Company
CD3	17A2	eBioscience
CD5	53-7.3	eBioscience
CD19	1D3	eBioscience
B220	RA3-6B2	eBioscience
CD11b	M1/70	eBioscience
Gr-1	RB6-8C5	eBioscience
Ter119	TER-119	eBioscience
FcϵRI	MAR	eBioscience
CD127	A7R34	eBioscience
NKp46	29A1.4	eBioscience
ICOS	C398.4	eBioscience
KLRG1	2F1	eBioscience
CCR6	29-2L17	eBioscience
IL-13	eBio13A	eBioscience
IFNγ	XMG1.2	eBioscience
CD45	30-F11	Invitrogen
RORγt	AFKJS-9	eBioscience
CD90.2	29A1.4	eBioscience
IL-5	TRFK5	BD
IL-17A	PAJ-17R	eBioscience
Ly6C	HK1.4	eBioscience
F4/80	T45-2342	eBioscience
Siglec-F	REA798	Miltenyi
CCR3	J073E5	Biolegend
NK1.1	PK136	Biolegend

### *In Vivo* Murine Faecal Microbiota Transplant Treatment

Faecal content extracted from the caecum of TRUC mice (31, 34) was reconstituted in sterile PBS 25% glycerol prior to storage at -80°C. Mice were orally gavaged with 200 μl aliquots of this faecal solution and sacrificed after 3 weeks.

### Statistics

Results are expressed as mean ± SEM. Data were analyzed using Student’s t-test or Mann-Whitney U test, as appropriate, using GraphPad Prism 5.0 (GraphPad Inc., USA). ns: non-significant; *p < 0.05; **p < 0.01; ***p <0.001; ****p <0.0001.

### Study Approval

Animal experiments were performed in accredited facilities in accordance with the UK Animals (Scientific Procedures) Act 1986 (Home Office Licence Numbers PPL: 70/6792, 70/8127, and 70/7869). Mice for studies on STAT1 and STAT4 were bred at the animal facility of the Institute of Animal Breeding and Genetics, University of Veterinary Medicine Vienna according to the guidelines of the Federal Ministry of Science, Research and Economy section 8ff of the Animal Science and Experiments Act, Tierversuchsgesetz [TVG], BMWF-68.205/0068-WF/V/3b/2015.

## Results

### T-Bet Control ILC3 Cellularity

**Figure 1:**
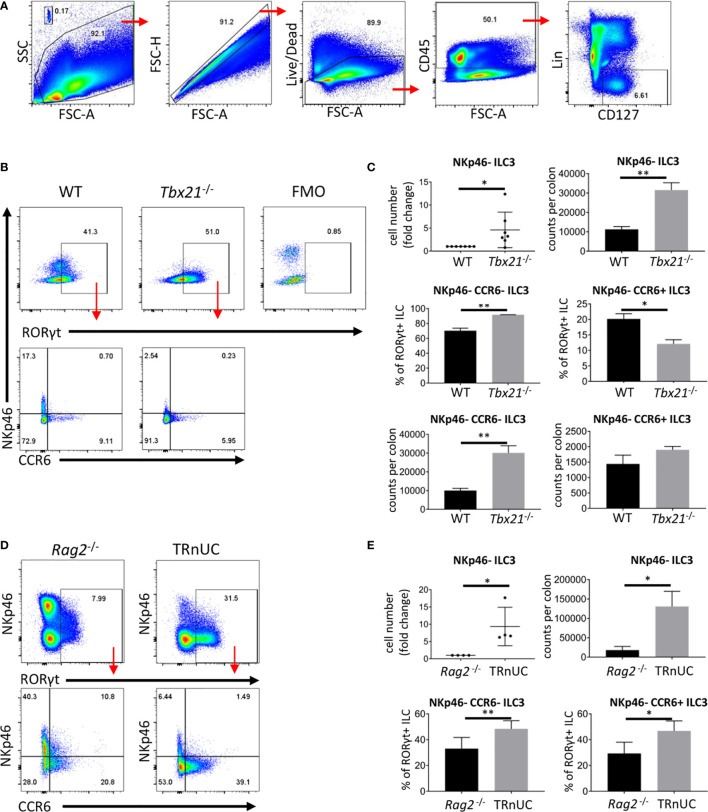
T-bet controls cellularity of intestinal NKp46^-^ ILC3 cLP. ILC of untreated mice were isolated for flow cytometry analysis. **(A)** ILC were gated as live CD45^+^ Lin^-^ CD127^+^ leukocytes. **(B)** NKp46^-^, CCR6^+^ and double-negative ILC3 in C57BL/6 and C57BL/6-background *Tbx21* ^-/-^ mice were analyzed as live CD45^+^ Lin^-^ CD127^+^ RORγt^+^ leukocytes. **(C)** Cell number fold change and counts per colon of total NKp46-negative ILC3 and percentage from RORγt^+^ ILC and counts per colon of NKp46^-^ CCR6^-^ and CCR6^+^ ILC3 are shown. **(D)** NKp46^-^, CCR6^+^ and double-negative cLP ILC3 from *Rag2* ^-/-^ and TRnUC mice were analyzed as live CD45^+^ Lin^-^ CD127^+^ RORγt^+^ leukocytes. **(E)** Cell number fold change and counts per colon of total NKp46-negative ILC3 and percentage of CCR6^+^ and NKp46^-^ CCR6^-^ ILC3 from RORγt^+^ ILC are shown. Data shown are representative of a minimum of 7 **(B, C)** or 4 **(D, E)** biological replicates.

We have reported that immunocompetent *Tbx21*-deficient mice do not develop spontaneous colitis (30). *Tbx21*-deficient mice lack ILC1 and NKp46^+^ ILC3, hence, we addressed the functionality of NKp46^-^ ILC3, which may also have a pathogenic role in colitis. For these analyses we define ILC as live CD45^+^ Lin^-^ CD127^+^ leukocytes (**Figure 1A**). Surprisingly, we detected an approximate 3-fold greater population of cLP NKp46-negative ILC3 in *Tbx21* ^-/-^ C57BL/6 mice in comparison to wild type (WT) mice (**Figures 1B, C**). Within this NKp46^-^ ILC3 population, there is a greater cellularity of NKp46^-^CCR6^-^ (double-negative) ILC3, but no significant difference in the cellularity of CCR6^+^ ILC3. Non-colitic *Rag2* ^-/-^x*Tbx21* ^-/-^ (TRnUC) mice also showed an enhanced cellularity of cLP NKp46^-^ ILC3 with a greater abundance of both NKp46^-^ CCR6^-^ and CCR6^+^ ILC3 (**Figures 1D, E**). Similar to cLP ILC3, there was a greater cellularity of small intestinal (SI) LP NKp46^-^ ILC3 in TRnUC mice and among these, the CCR6^+^ ILC3 population size was increased significantly (**Supplementary Figures 1A, B**). For these analyses, TRnUC mice on a BALB/c background were chosen because *Rag2* ^-/-^x*Tbx21* ^-/-^ mice on this background appear to be more prone to colitis than C57BL/6 background *Rag2* ^-/-^x*Tbx21* ^-/-^ mice (35). Overall, these observations support the notion that T-bet controls the cellularity of NKp46^-^ ILC3.

**Figure 2:**
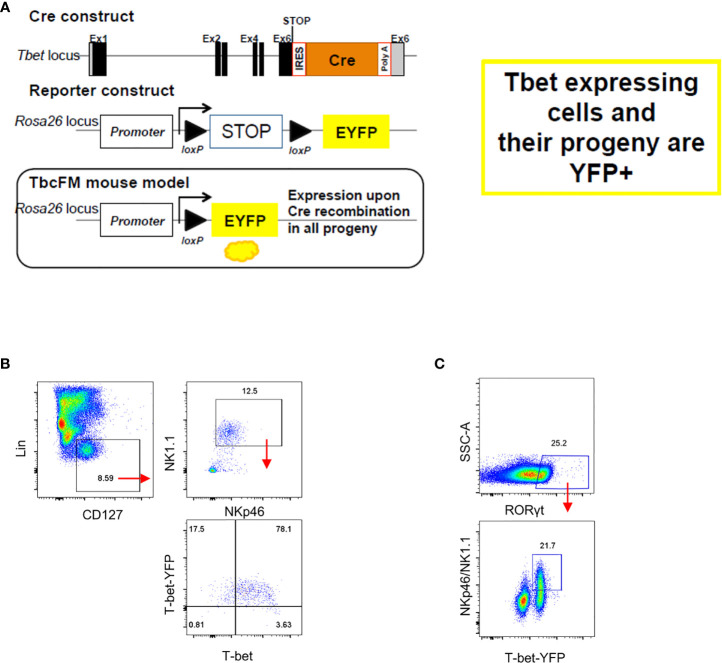
NKp46^-^ ILC3 have fate mapper expression of T-bet. Intestinal lamina propria ILC were isolated for flow cytometry analysis. **(A)** See method section for further details on the design. **(B)** T-bet^FM^ and T-bet expression in live CD45^+^ Lin^-^ CD127^+^ NKp46^+^ NK1.1^+^ cLP ILC and **(C)** T-bet^FM^ expression in live CD45^+^ Lin^-^ CD127^+^ NKp46^+^ and NKp46^-^ SI LP ILC3 are illustrated. Data shown are representative of one experiment **(B)** or 2 **(C)** replicates.

Enhanced cellularity of NKp46^-^ CCR6^-^ ILC3 in *Tbx21*-deficient mice supported a previous report indicating that these cells are the precursors of NKp46^+^ ILC3 (2). Hence, the lack of T-bet could result to a developmental blockade and the accumulation of NKp46^-^ CCR6^-^ ILC3. An interlinkage of NKp46^+^ ILC3 and NKp46^-^ CCR6^-^ ILC3 was also highlighted in another study reporting that NKp46^+^ ILC3 can lose NKp46 expression (36). In order to detect NKp46^-^ CCR6^-^ ILC3 with a history of T-bet expression, we generated a mouse model that expresses Cre-recombinase under the expression of the *Tbx21* endogenous promoter by inserting an IRES-Cre cassette downstream of the *Tbx21* stop codon (**Figure 2A**). This T-bet^Cre^ mouse was then bred to the Rosa26-lox-stop-lox YFP mouse (Rosa26^YFP/+^) (37) to generate the T-bet^Cre/+^x*Rosa26* ^YFP/+^ fate-mapper mouse (T-bet^FM^). As expected, cLP ILC1 defined as NKp46^+^NK1.1^+^T-bet^+^ were found to be T-bet fate mapper positive (T-bet^FM+^) (**Figure 2B**), confirming the functionality of the model as these cells have previously been shown to express T-bet. Interestingly, T-bet^FM+^ NKp46^-^ SI LP ILC3 were detected supporting the notion that these cells have potential to express this transcription factor (**Figure 2C**).

**Figure 3:**
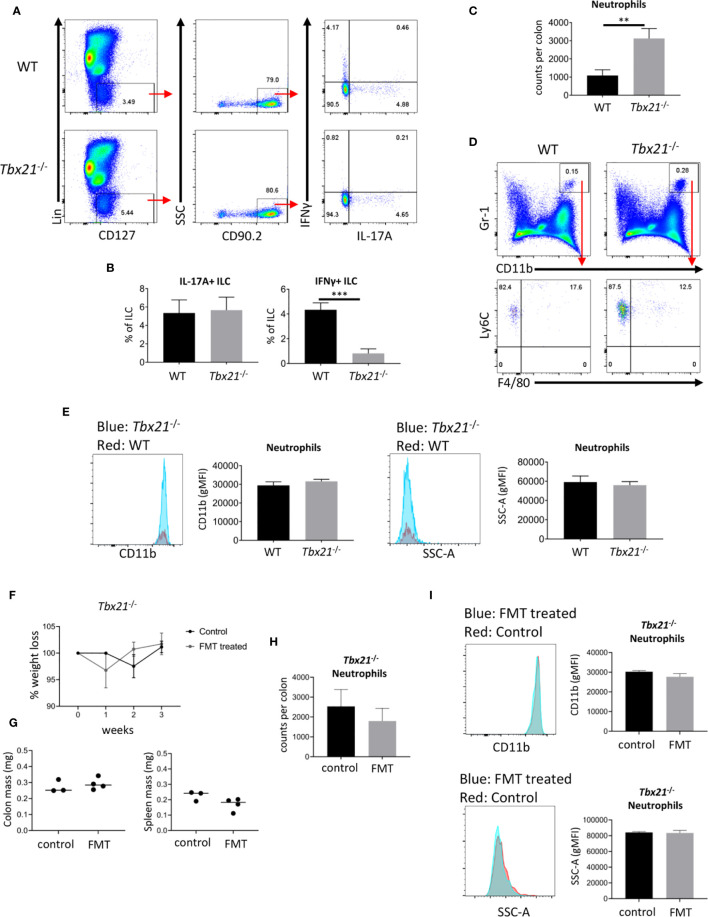
T-bet deficiency promotes intestinal neutrophilia. Leukocytes were isolated from the colonic lamina propria of untreated WT and *Tbx21* ^-/-^ mice for flow cytometry analysis. **(A, B)** IL-17A and IFNγ expression in live CD45^+^ Lin^-^ CD127^+^ CD90.2^+^ leukocytes after a 4 h stimulation with PMA and ionomycin was analyzed and statistical analyses are shown. **(C, D)** CD11b^+^ Gr1^+^ Ly6C^+^ F4/80^-^ neutrophils were analyzed from a live CD45^+^ cLP leukocyte population and neutrophil counts per colon are shown. **(E)** CD11b geometric median fluorescence intensity (gMFI) and granularity (SSC-A gMFI) were determined for WT and *Tbx21* ^-/-^ cLP neutrophils. **(F–I)** *Tbx21* ^-/-^ mice received a fecal transplant with pathogenic microbes derived from TRUC mice (31). **(F)** Changes in body weights were monitored on a weekly basis in *Tbx21* ^-/-^ mice upon FMT and *Tbx21* ^-/-^ control mice. Colon and spleen mass **(G)** and cLP neutrophil cellularity **(H)** were determined 3 weeks upon FMT treatment. **(I)** CD11b gMFI and SSC-A gMFI in cLP neutrophils 3 weeks upon FMT treatment are illustrated. Data shown are representative of 3 biological replicates.

To further evaluate the ILC3 phenotype, we analyzed the cytokine profiles of CD127^+^ ILC in *Tbx21* ^-/-^ and WT mice. As expected CD127^+^ ILC from *Tbx21* ^-/-^ mice produced very low amounts of IFNγ, but surprisingly there was no altered expression of IL-17A on a per cell basis in the same mice (**Figures 3A, B**). However, due to the greater cellularity of CD127^+^ ILC in the intestine of mice lacking T-bet (13) we anticipate more ILC expressing IL-17A. Hence, we aimed to investigate whether T-bet also controls the cellularity of cLP neutrophils (**Figures 3C, D**). In line with the greater cellularity of NKp46^-^ ILC3 in *Tbx21* ^-/-^ mice, there was indeed a greater neutrophilia in these mice. These neutrophils had an unaltered level of CD11b expression and granularity measured as SSC-A (**Figure 3E**) indicating that these neutrophils were not activated. We aimed to determine whether these neutrophils can be activated with a pathogenic microbiota. In order to test this *Tbx21* ^-/-^ mice received an oral gauvage injection of fecal microbes derived from colitic *Rag2* ^-/-^x*Tbx21* ^-/-^ (TRUC) mice (31, 34). These *Tbx21* ^-/-^ mice did not show weight abnormalities during the course of 3 weeks upon fecal microbial transplant (FMT) in comparison to *Tbx21* ^-/-^ control mice (**Figure 3F**). Furthermore, the mass of colon and spleen did not show a significant difference due to FMT 3 weeks after the treatment (**Figure 3G**). FMT did also not result in altered cLP neutrophilia or cLP neutrophil activation detected by CD11b expression and granularity at this time point (**Figures 3H, I**). Hence, *Tbx21* ^-/-^ mice appeared to be resilient to spontaneous colitis driven by the pathogenic microbes used.

We have shown that untreated *Tbx21* ^-/-^ mice have a greater cellularity and activation of ILC2 (13). Hence, IL-5-producing ILC2 may promote an immune response counteracting to a more pathogenic response driven by ILC3 in naïve *Tbx21*-deficient mice (13). To explore this further we analyzed cLP eosinophilia in *Tbx21* ^-/-^ mice, but detected no significant difference to WT mice (**Supplementary Figure 2**).

### T-Bet Deficient NKp46^-^ ILC3 Have Enhanced Expression of RORγt and CD127

**Figure 4:**
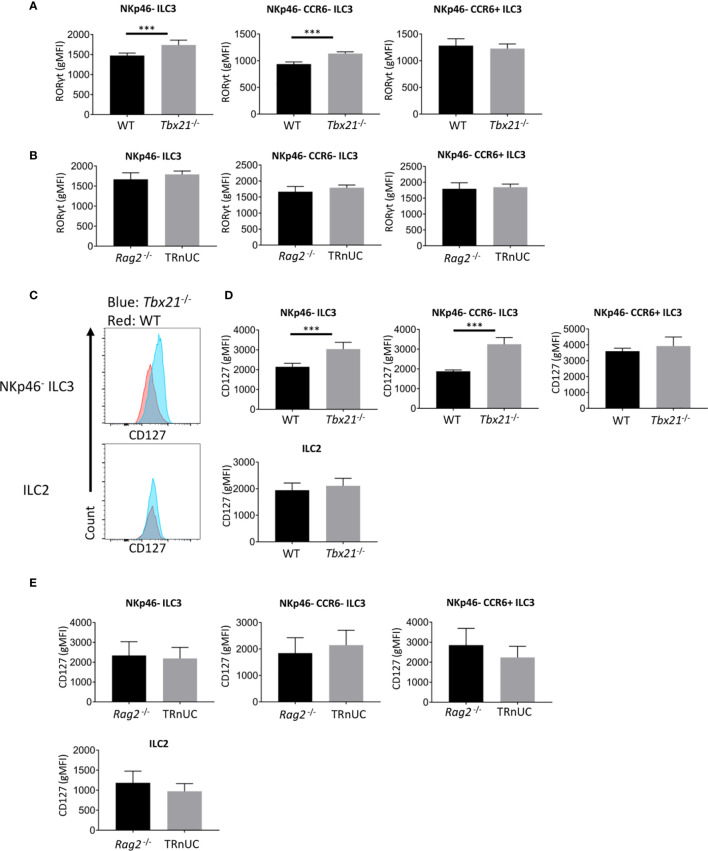
T-bet deficiency promotes RORγt and CD127 expression by cLP ILC3. ILC were isolated from the colonic lamina propria of C57BL/6 and C57BL/6-background *Tbx21* ^-/-^, and BALB/c-background *Rag2* ^-/-^ and TRnUC mice for flow cytometry analysis of RORγt and CD127 expression. RORγt gMFI expression in total NKp46^-^, CCR6^+^ and double-negative ILC3 from **(A)** WT and *Tbx21* ^-/-^ and **(B)** *Rag2* ^-/-^ and TRnUC mice was analyzed within the live CD45^+^ Lin^-^ CD127^+^ RORγt^+^ leukocyte population. **(C)** CD127 surface expression in total NKp46-negative ILC3 and ILC2 defined as KLRG1^+^ ICOS^+^ CD127^+^ ILC is illustrated with flow cytometry histograms. Statistical analyses of CD127 surface expression (gMFI) on total NKp46^-^, CCR6^+^ and double-negative ILC3 and ILC2 from **(D)** WT and *Tbx21* ^-/-^ and **(E)** *Rag2* ^-/-^ mice are presented. Data shown are representative of a minimum of 7 **(A–D)** or 4 **(E)** biological replicates.

Strikingly, the finding of enhanced cellularity of NKp46^-^ ILC3 in *Tbx21* ^-/-^ mice correlated with enhanced RORγt expression in total NKp46^-^ CCR6^-^ ILC3, but not in CCR6^+^ ILC3 (**Figure 4A**). In contrast, in TRnUC mice RORγt expression was only enhanced in SI LP NKp46^-^ CCR6^-^ ILC3, but not SI LP CCR6^+^ ILC3 or cLP ILC3 (**Supplementary Figure 3**, **Figure 4B**).

Previously, we have also reported that *Tbx21* deficiency causes greater expression of the α chain of IL-7R (CD127) on total CD127^+^ ILC in the intestine (13). In the current study we can now pinpoint that within this *Tbx21*-deficient population cLP NKp46^-^ CCR6^-^ ILC3, but not CCR6^+^ ILC3 or ILC2 express more CD127 (**Figures 4C, D**). This was again in contrast to TRnUC mice as cLP and SI LP ILC3 and ILC2 in these mice did not display an altered CD127 expression in comparison to *Rag2* ^-/-^ mice (**Figure 4E**, **Supplementary Figures 3B, C**). Mechanistically, we have reported previously that T-bet binds to the *Cd127* locus in Th1 cells polarized *in vitro* (31, 38). Hence, it appears that T-bet is a regulator of *Cd127* and *Rorc* (encoding RORγt) expression at the transcriptional level which may be factors limiting the cellularity of NKp46^-^ CCR6^-^ ILC3 in Rag-sufficient mice.

### Mice Deficient in IFNγ, IL-27Rα, STAT1, or STAT4 Have Unaltered Cellularity of NKp46^-^ ILC3

**Figure 5:**
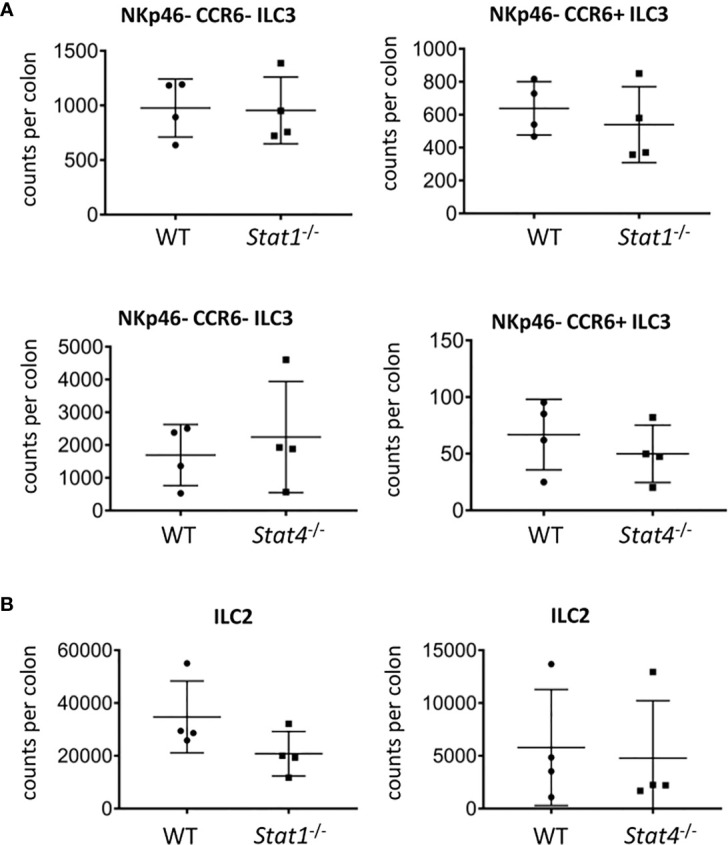
STAT1 and STAT4 deficiency does not promote ILC3 cellularity. cLP ILC were isolated from mice for flow cytometry analysis. Counts per colons of **(A)** NKp46^-^CCR6^-^ and CCR6^+^ ILC3 and **(B)** ILC2 in WT, *Stat*1^-/-^ and *Stat4* ^-/-^ mice are shown. Data shown are representative of 4 biological replicates.

We further aimed to identify mediators upstream of T-bet that may be involved in limiting the cellularity of NKp46^-^ ILC3. Surprisingly, there was no alteration in the cellularity of NKp46^-^ CCR6^-^ and CCR6^+^ cLP ILC3 and no change in RORγt and CD127 expression in ILC3 derived from STAT1 (*Stat1* ^-/-^) or STAT4 (*Stat4* ^-/-^) deficient mice (**Figure 5A**, **Supplementary Figures 4C, D**). This observation was confirmed by the analysis of the same parameters in those ILC3 from mice deficient of IFNγ (*Ifng* ^-/-^) or IL-27Rα (*Il27RA* ^-/-^), both of which cause signaling events through STAT1 (**Supplementary Figures 4A–D**). Furthermore, the absence of STAT4 but not STAT1 in the germline did result in significantly altered IL-17A expression in CD127^+^ CD90.2^+^ cLP ILC (**Supplementary Figures 4E, F**).

T-bet-deficient mice have a greater cellularity of cLP ILC2 (13). In order to correlate with these data, we analyzed ILC2 abundance in *Stat1* ^-/-^ and *Stat4* ^-/-^ mice. As observed with NKp46-negative ILC3, the cellularity of cLP ILC2 did not alter in mice deficient of either STAT1 or STAT4 and CD127 expression levels in these cells did also not change (**Figure 5B**, **Supplementary Figure 5A**). Furthermore, CD127 expression was not altered in cLP ILC2 from *Il27RA* ^-/-^ mice (**Supplementary Figure 5A**). Interestingly, the potency of CD127^+^ CD90.2^+^ cLP ILC to co-produce IL-13 and IL-5 was reduced in *Stat1* ^-/-^ but not in *Stat4* ^-/-^ (**Supplementary Figure 5B**). Overall, STAT1 and STAT4 did not appear to play a critical role in in controlling the cellularity of the NKp46-negative ILC3 populations and ILC2 in the cLP (**Figure 5**).

## Discussion

There is currently no effective drug to cure IBD, and there is an urgent need to identify novel strategies of treatment. We and others have identified T-bet to be central to drive the severity of colitis in human and mice. However, targeting T-bet for instance using a small molecule inhibitor as has been tested for RORγt (39, 40) requires more detailed understanding of the functional role of ILC3 in the absence of T-bet. The relevance of this has been demonstrated by the protective role of T-bet in Rag-deficient mice infected with *H. thyphlonius* (31, 34). These data indicate that IL-17A^+^ ILC, which could be ILC3 or inflammatory ILC2 (41), can drive colitis in the absence of T-bet and adaptive immune responses, such as regulatory T cells and sIgA, sIgM, and sIgG production.

In this study, we report that T-bet deficiency results in a very significantly increased cellularity of NKp46-negative ILC3 in Rag-deficient and Rag-sufficient naïve mice. Previously, we reported an approximate 2-fold increase in cLP ILC2 in T-bet-deficient mice (13). Importantly, greater cellularity of ILC3 in *Tbx21* ^-/-^ mice correlated with enhanced neutrophilia in Rag-sufficient *Tbx21* ^-/-^ mice. However, the activation of these neutrophils was not altered by T-bet-deficiency which might be due to an intact epithelial barrier preventing microbes to infiltrate the tissue. Overall, T-bet deficiency caused increased NKp46^-^ CCR6^-^ ILC3 and ILC2 cellularity in Rag-sufficient mice, but not at the cost of spontaneous colitis as observed in *H. thyphlonius*-infected *Rag2* ^-/-^x*Tbx21* ^-/-^ mice (31, 34).

A study by Klose et al. (2) suggested that NKp46^-^ CCR6^-^ ILC3 may be precursor cells of NKp46^+^ ILC3 in the intestine. Considering these data, it may be possible that the accumulation of NKp46^-^ CCR6^-^ ILC3 in T-bet-deficient mice is a consequence of the inhibited further differentiation.

Mechanistically, greater cellularity of NKp46^-^ CCR6^-^ ILC3 was correlated to greater expression of IL-7Rα and RORγt in Rag-sufficient mice. The same effect was not observed in Rag-deficient mice suggesting that in these mice those factors are unlikely driving factors of NKp46^-^ ILC3 cellularity in the absence of T-bet. In contrast to NKp46^-^ CCR6^-^ ILC3, an enhanced CD127 expression was not observed in CCR6^+^ ILC3 and ILC2 in *Tbx21*-deficient mice pointing to an intrinsic T-bet-dependent CD127 regulation pathway in NKp46^-^ CCR6^-^ ILC3. Interestingly, we observed that T-bet can bind to the *Cd127* gene locus in CD4 T cells which may indicate that T-bet functions as a repressor of CD127 expression (31, 38). In CD4 T cells, T-bet is known to inhibit transcription of *Rorc* (encoding RORγt), and this may be also a critical intrinsic mechanism for enhanced RORγt expression in *Tbx21*-deficient NKp46^-^ CCR6^-^ ILC3 (42). Interestingly, T-bet-dependent regulation of RORγt and CD127 in NKp46^-^ CCR6^-^ ILC3 was only observed in Rag-sufficient C57BL/6 and not Rag-deficient BALB/c mice. Despite of this, T-bet in these Rag-deficient mice regulated cellularity of NKp46^-^ CCR6^-^ ILC3 and CCR6^+^ ILC3. These data indicate that T-bet may regulate the cellularity of NKp46^-^ CCR6^-^ ILC3 *via* further mechanisms not controlled by CD127 and RORγt expression in ILC.

We also aimed to reveal potential further signaling pathways that enhance T-bet-driven effects in NKp46-negative ILC3 and ILC2. Both IFNγ and IL-27 are known to promote T-bet expression and at least pulmonary ILC3 have been reported to express the receptors for both cytokines (43–46), however, we report here that the deficiency of neither of these mediators and STAT1 resulted in the enhanced cellularity of NKp46-negative ILC3 or ILC2. IFNγ signals through a STAT1 homodimer, while a STAT1:STAT3 heterodimer is employed downstream of the IL-27 receptor (45, 47). Furthermore, STAT4 as an important signal transmitter downstream of the IL-12 receptor regulating T-bet expression (48) did not appear to have a crucial role in the cellularity of NKp46-negative ILC3 and ILC2 as well. In addition, neither STAT1 nor STAT4 signaling events appeared to control CD127 and RORγt expression in cLP NKp46-negative ILC3. Overall, this study suggests that RORγt and IL-7R are plausible targets of T-bet to limit the cellularity of NKp46^-^ CCR6^-^ ILC3 and neutrophils in the colonic lamina propria of immunocompetent mice. This is of critical importance to evaluate potential side effects of T-bet targeted treatment of IBD.

## Data Availability Statement

The original contributions presented in the study are included in the article/**Supplementary Material**. Further inquiries can be directed to the corresponding author.

## Ethics Statement

The animal study was reviewed and approved by UK Animals (Scientific Procedures) Act 1986. Written informed consent was obtained from the owners for the participation of their animals in this study.

## Author Contributions

Study concept and design J-HS, JH, GL, NP, BS, acquisition of data J-HS, KM, DH, JL, JN, HH, data analysis and interpretation J-HS, KM, JL, JN, NP, BS, GL, obtained funding GL, BS, NP, drafting of manuscript J-HS, study supervision GL. All authors contributed to the article and approved the submitted version.

## Funding

This study was supported by grants awarded by the Wellcome Trust (GL, 091009) and the Medical Research Council (GL, MR/M003493/1; GL, MR/K002996/1; JH, MR/K002996/1). Research was also supported by the National Institute for Health Research (NIHR) Biomedical Research Centre at Guy’s and St Thomas and King’s College London (GL), the Austrian Science Fund (BS, FWF, SFB-F6101), the Welcome Trust (NP, JL, WT101159) and the NIHR Imperial Biomedical Research Center (NP, JL, BRC). JN acknowledges a RCUK/UKRI Rutherford Fund fellowship (MR/R024812/1). The views expressed are those of the author(s) and not necessarily those of the NHS, the NIHR, or the Department of Health.

## Conflict of Interest

The authors declare that the research was conducted in the absence of any commercial or financial relationships that could be construed as a potential conflict of interest.

## Supplementary Material

Supplementary Figure 1 T-bet deficiency promotes *Rag2* ^-/-^ SI LP NKp46^-^ ILC3 cellularity. ILC were isolated from the SI LP of mice for flow cytometry analysis. **(A)** NKp46^-^, CCR6^+^, and double-negative ILC3 in *Rag2* ^-/-^ and TRnUC mice were analyzed as live CD45^+^ Lin^-^ CD127^+^ RORγt^+^ leukocytes. **(B)** Cell number fold change, counts per colon of total NKp46-negative ILC3 and percentage of NKp46^-^ CCR6^-^ ILC3 and CCR6^+^ ILC3 within the RORγt^+^ ILC population are shown. Data shown are representative of 4 biological replicates.

Supplementary Figure 2 Eosinophilia is not affected in T-bet deficient mice. cLP eosinophils were isolated for flow cytometry analysis. **(A, B)** Surface expression of CD11b, Siglec-F, CCR3, and Ly6C in live CD45^+^ cLP leukocytes from WT and *Tbx21* ^-/-^ mice and statistical analysis of eosinophil percentage among CD45^+^ leukocytes are shown. Data shown are representative of 3 biological replicates.

Supplementary Figure 3 RORγt and CD127 expression is not altered in *Rag2* ^-/-^ x *Tbx21* ^-/-^ SI LP NKp46^-^ ILC3. ILC were isolated from the SI LP of *Rag2* ^-/-^ and TRnUC mice for flow cytometry analysis. **(A)** RORγt and **(B)** CD127 gMFI expression in total NKp46-negative ILC3, NKp46^-^ CCR6^-^ ILC3, and CCR6^+^ ILC3 are shown. **(C)** CD127 gMFI expression in ILC2 is shown. Data shown are representative of 4 biological replicates.

Supplementary Figure 4 Analysis of cLP ILC3 from mice deficient of IFNγ, IL-27Rα, STAT1, or STAT4. cLP ILC3 were isolated for flow cytometry analysis. NKp46^-^ CCR6^-^ and CCR6^+^ cLP ILC3 from C57BL/6 and either *Ifng* ^-/-^, *Il27RA* ^-/-^, *Stat1* ^-/-^, or *Stat4* ^-/-^ mice were analyzed as live CD45^+^ Lin^-^ CD127^+^ RORγt^+^ leukocytes. **(A)** ILC3 in *Ifng* ^-/-^ and *Il27RA* ^-/-^ mice and **(B)** the percentage of NKp46^-^ CCR6^-^ and CCR6^+^ ILC3 of total ILC3 are shown. **(C)** RORγt and **(D)** CD127 gMFI expression in NKp46^-^ CCR6^-^ and CCR6^+^ ILC3 in WT and either *Ifng* ^-/-^, *Il27RA* ^-/-^, *Stat1* ^-/-^, or *Stat4* ^-/-^ mice are illustrated. IL-17A expression in live CD45^+^ Lin^-^ CD127^+^ CD90.2^+^ ILC from **(E)** *Stat1* ^-/-^ or **(F)** *Stat4* ^-/-^ mice are shown. Data shown are representative of a minimum of 3 biological replicates.

Supplementary Figure 5 cLP ILC2 analysis in *Stat1* ^-/-^ and *Stat4* ^-/-^ mice. cLP ILC were isolated for flow cytometry analysis. **(A)** CD127 gMFI expression in live CD45^+^ Lin^-^ CD127^+^ ICOS^+^ KLRG1^+^ ILC from *Stat1* ^-/-^, *Il27RA* ^-/-^, or *Stat4* ^-/-^ mice. **(B)** Percentage of IL-13 and IL-5 co-expression in live CD45^+^ Lin^-^ CD127^+^ CD90.2^+^ ILC from *Stat1* ^-/-^ or *Stat4* ^-/-^ mice. Data shown are representative of 4 biological replicates.
