# Supplementary figures and images for: T-Bet Controls Cellularity of Intestinal Group 3 Innate Lymphoid Cells
Source: Front Immunol. 2021 Feb 2;11:623324. doi: 10.3389/fimmu.2020.623324 (PMC7884460; doi:10.3389/fimmu.2020.623324)

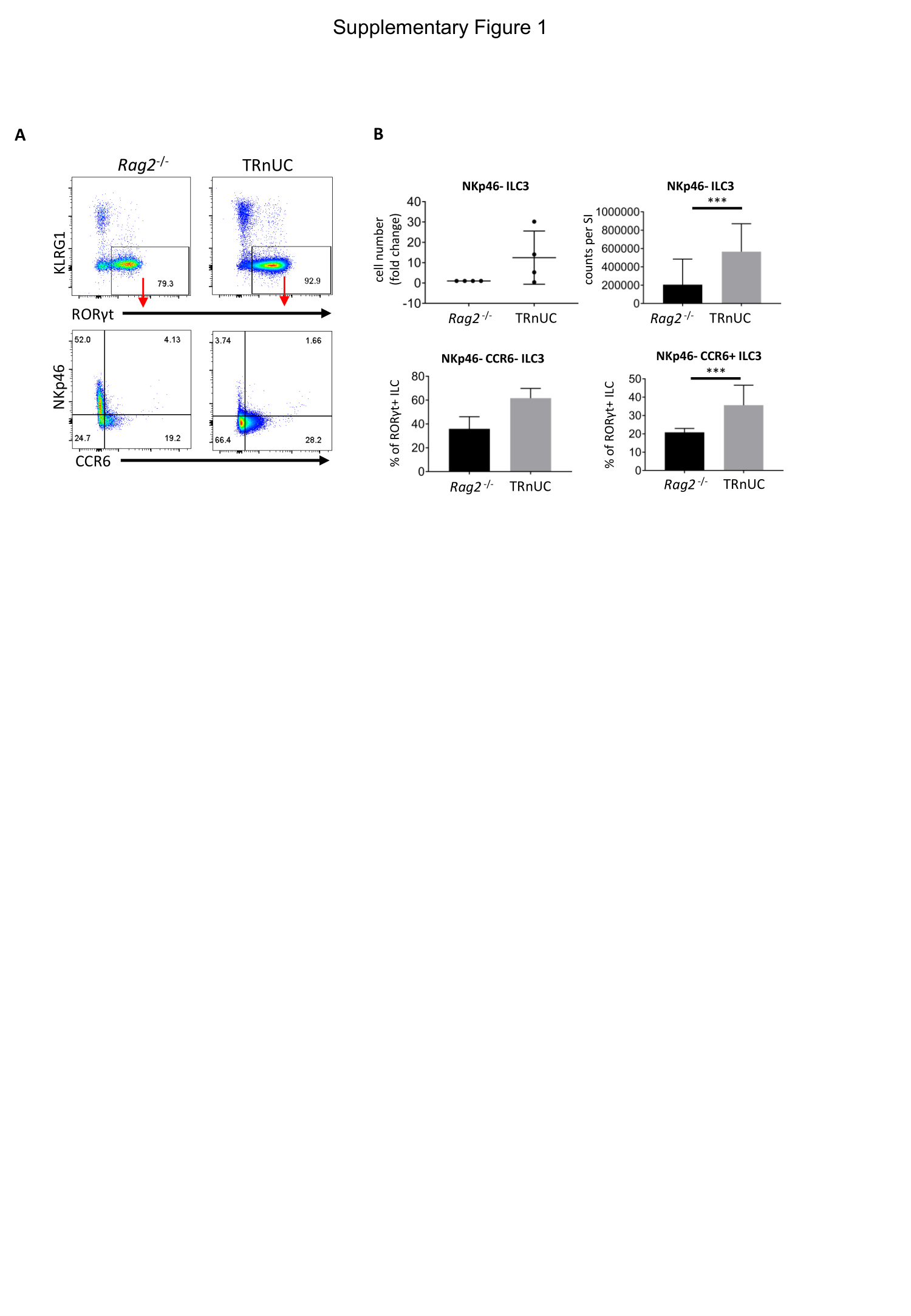

Supplement: Supplementary Figure 1 — T-bet deficiency promotes Rag2 -/- SI LP NKp46- ILC3 cellularity. ILC were isolated from the SI LP of mice for flow cytometry analysis. (A) NKp46-, CCR6+, and double-negative ILC3 in Rag2 -/- and TRnUC mice were analyzed as live CD45+ Lin- CD127+ RORγt+ leukocytes. (B) Cell number fold change, counts per colon of total NKp46-negative ILC3 and percentage of NKp46- CCR6- ILC3 and CCR6+ ILC3 within the RORγt+ ILC population are shown. Data shown are representative of 4 biological replicates. [file Image_1.tiff]

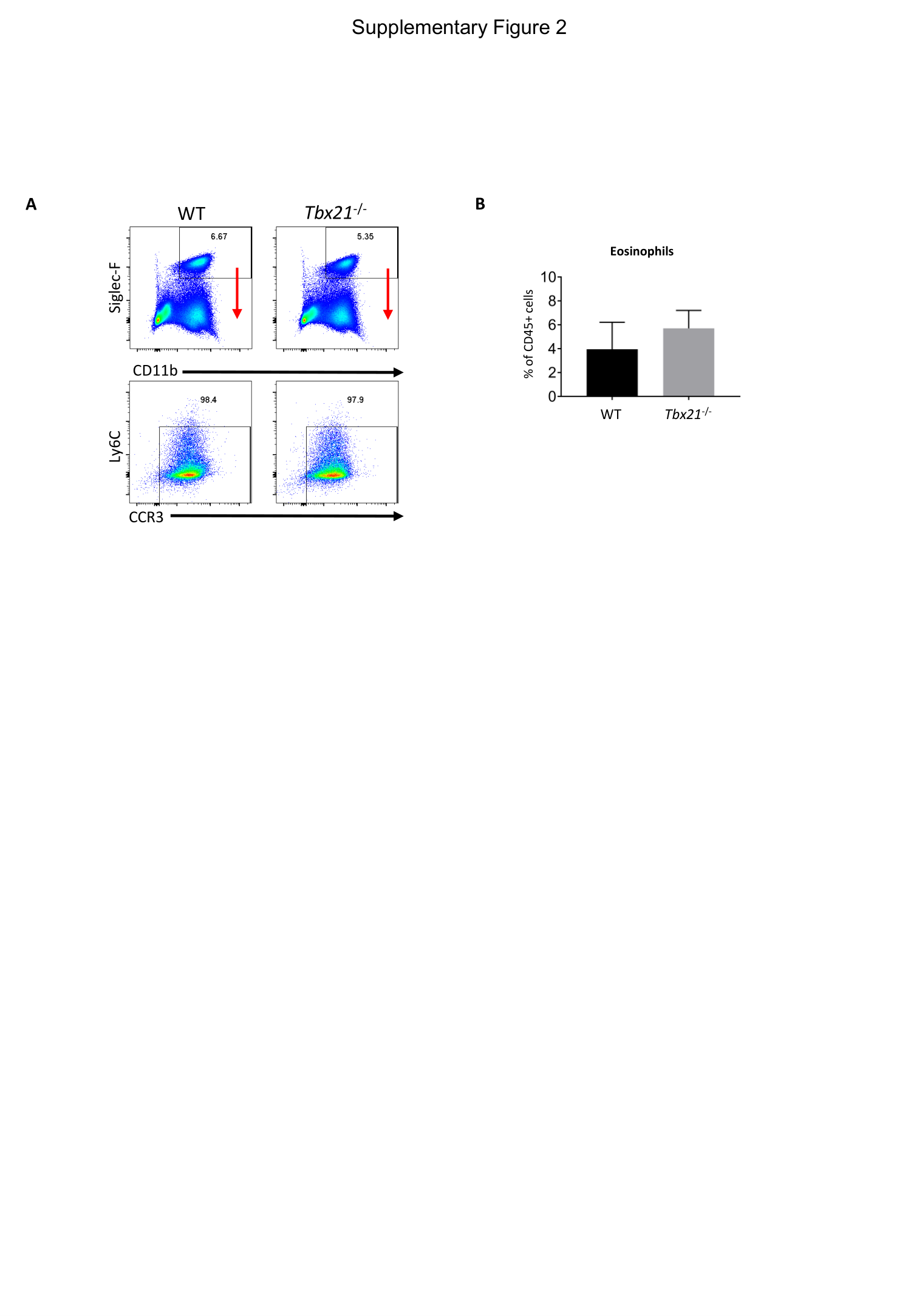

Supplement: Supplementary Figure 2 — Eosinophilia is not affected in T-bet deficient mice. cLP eosinophils were isolated for flow cytometry analysis. (A, B) Surface expression of CD11b, Siglec-F, CCR3, and Ly6C in live CD45+ cLP leukocytes from WT and Tbx21 -/- mice and statistical analysis of eosinophil percentage among CD45+ leukocytes are shown. Data shown are representative of 3 biological replicates. [file Image_2.tiff]

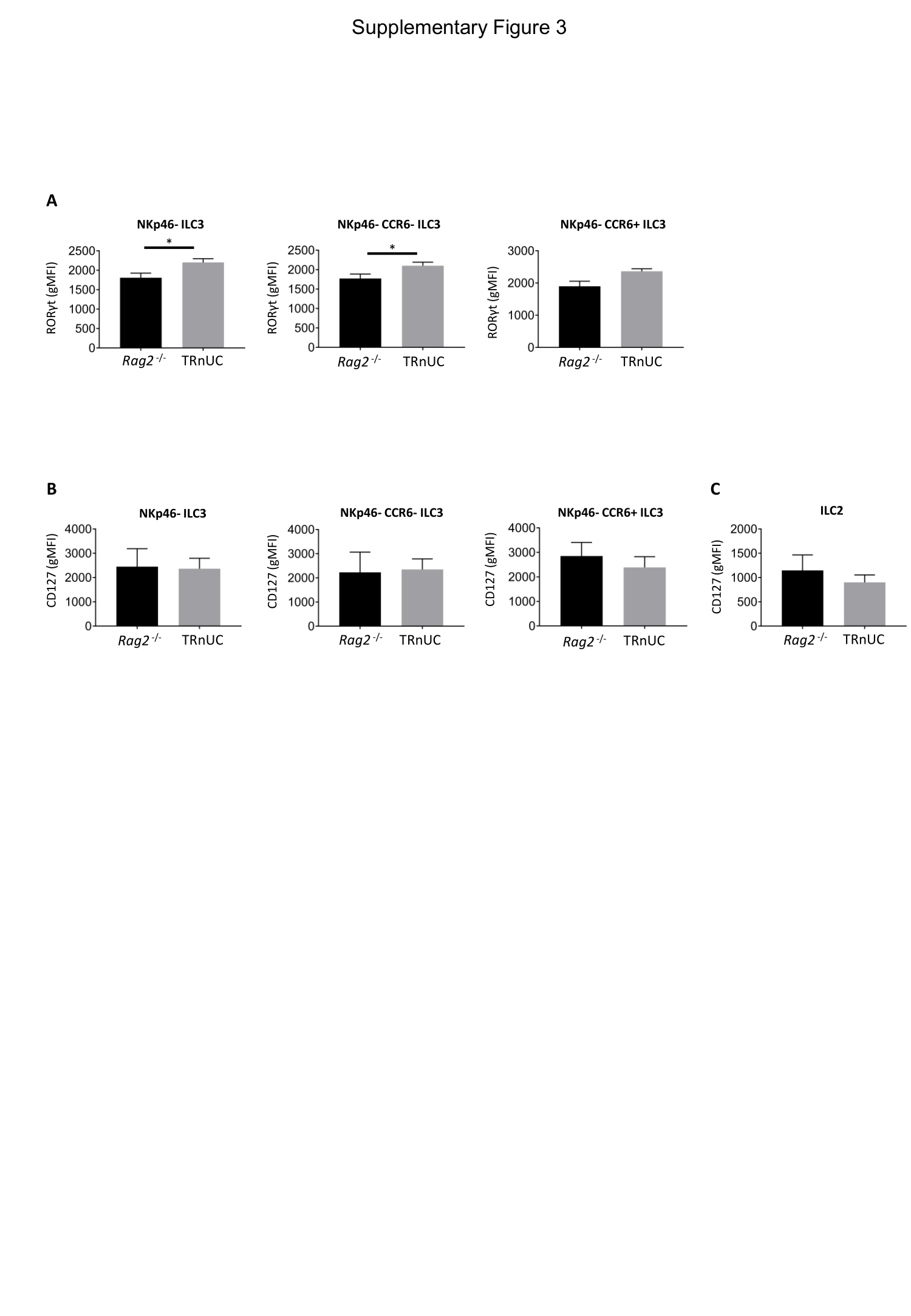

Supplement: Supplementary Figure 3 — RORγt and CD127 expression is not altered in Rag2 -/- x Tbx21 -/- SI LP NKp46- ILC3. ILC were isolated from the SI LP of Rag2 -/- and TRnUC mice for flow cytometry analysis. (A) RORγt and (B) CD127 gMFI expression in total NKp46-negative ILC3, NKp46- CCR6- ILC3, and CCR6+ ILC3 are shown. (C) CD127 gMFI expression in ILC2 is shown. Data shown are representative of 4 biological replicates. [file Image_3.tiff]

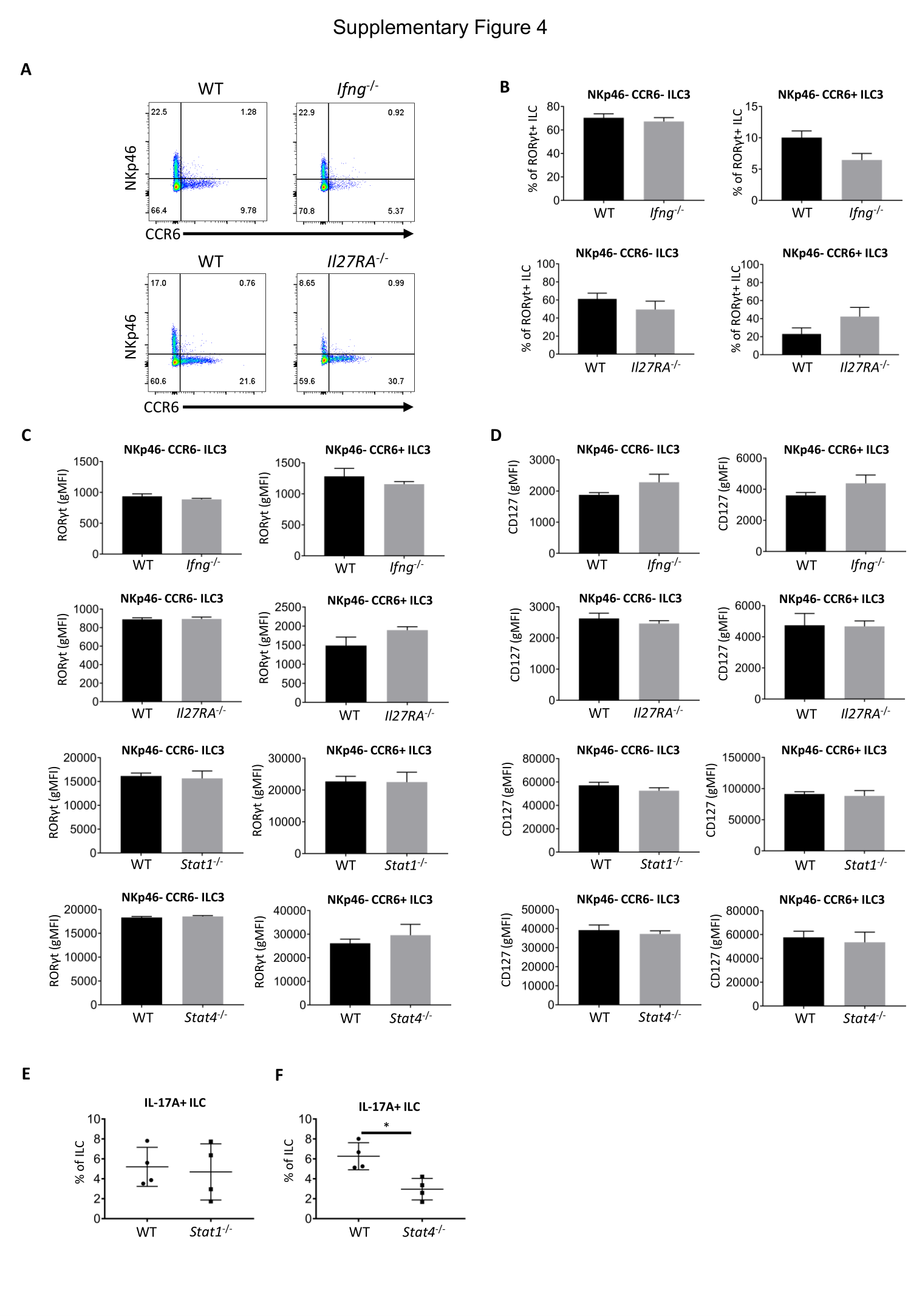

Supplement: Supplementary Figure 4 — Analysis of cLP ILC3 from mice deficient of IFNγ, IL-27Rα, STAT1, or STAT4. cLP ILC3 were isolated for flow cytometry analysis. NKp46- CCR6- and CCR6+ cLP ILC3 from C57BL/6 and either Ifng -/-, Il27RA -/-, Stat1 -/-, or Stat4 -/- mice were analyzed as live CD45+ Lin- CD127+ RORγt+ leukocytes. (A) ILC3 in Ifng -/- and Il27RA -/- mice and (B) the percentage of NKp46- CCR6- and CCR6+ ILC3 of total ILC3 are shown. (C) RORγt and (D) CD127 gMFI expression in NKp46- CCR6- and CCR6+ ILC3 in WT and either Ifng -/-, Il27RA -/-, Stat1 -/-, or Stat4 -/- mice are illustrated. IL-17A expression in live CD45+ Lin- CD127+ CD90.2+ ILC from (E) Stat1 -/- or (F) Stat4 -/- mice are shown. Data shown are representative of a minimum of 3 biological replicates. [file Image_4.tiff]

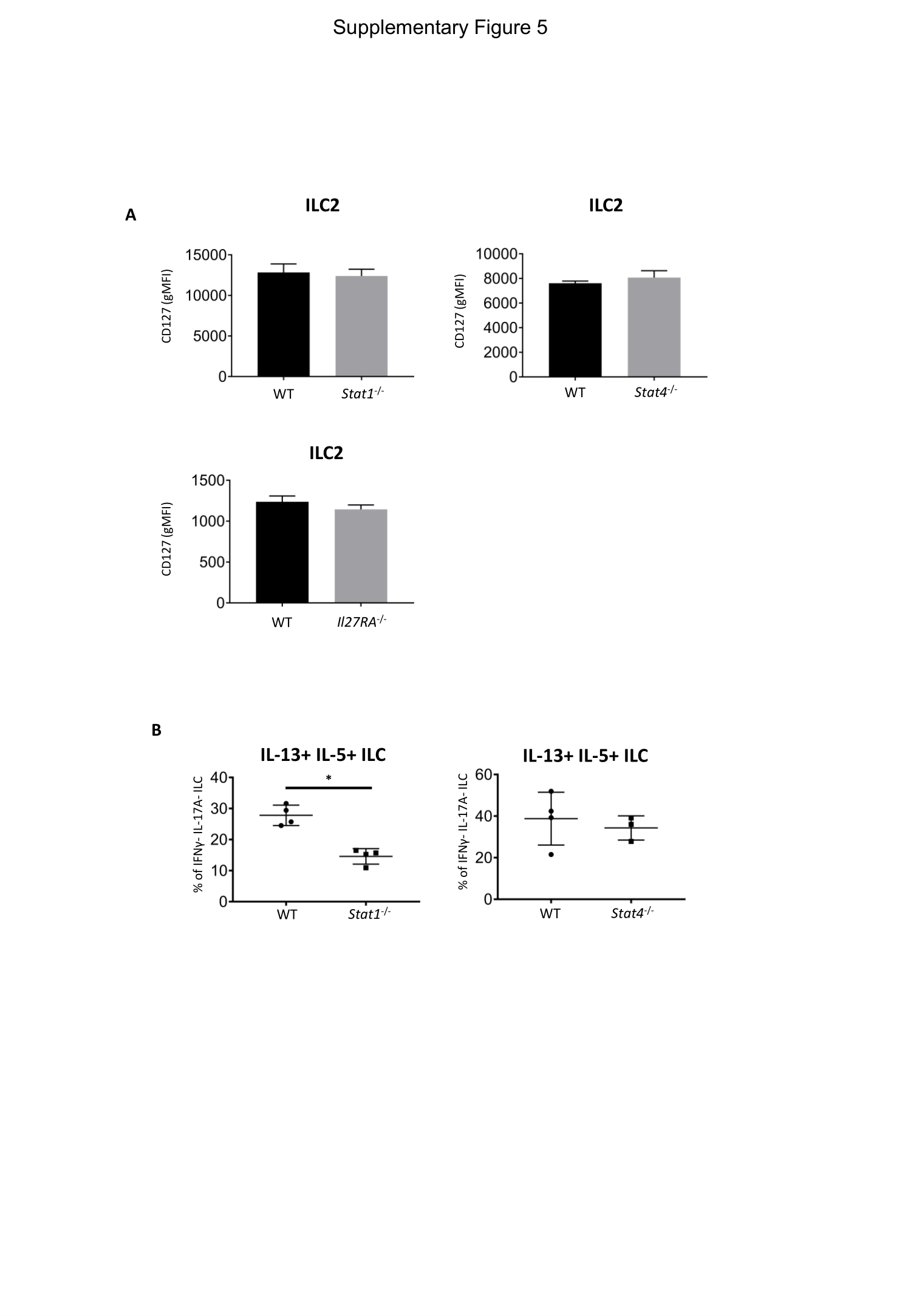

Supplement: Supplementary Figure 5 — cLP ILC2 analysis in Stat1 -/- and Stat4 -/- mice. cLP ILC were isolated for flow cytometry analysis. (A) CD127 gMFI expression in live CD45+ Lin- CD127+ ICOS+ KLRG1+ ILC from Stat1 -/-, Il27RA -/-, or Stat4 -/- mice. (B) Percentage of IL-13 and IL-5 co-expression in live CD45+ Lin- CD127+ CD90.2+ ILC from Stat1 -/- or Stat4 -/- mice. Data shown are representative of 4 biological replicates. [file Image_5.tiff]
